# Comprehensive analysis of the effects of the traditional stir-fry process on the dynamic changes of volatile metabolites in Hainan camellia oil

**DOI:** 10.1016/j.fochx.2024.101747

**Published:** 2024-08-17

**Authors:** Tengfei Xia, Zijun Xiong, Chunmei Wang, Xiuxiu Sun, Yeguang Chen, Jiali Chen, Huasha Qi, Heng Liang, Lang Zhang, Daojun Zheng

**Affiliations:** a Institute of Tropical Horticulture Research, Hainan, Academy of Agricultural Sciences, Haikou 571100, China; b Sanya Institute of Hainan Academy of Agricultural Sciences, Sanya 572025, China; c Institute of Tropical Bioscience and Biotechnology, Chinese Academy of Tropical Agricultural Sciences, Haikou 571101, China

**Keywords:** Traditional stir-fry process, *Camellia drupifera*, Hainan camellia oil, Volatile metabolites, GC × GC TOFMS, Network pharmacology approach

## Abstract

The traditional stir-fry process before pressing is crucial to manufacture Hainan camellia oil. To assess the effects of the stir-fry process on Hainan camellia oil, six samples across different stir-fry stages were analyzed. The stir-fry process modified odors, volatile metabolite profiles, and human health-promoting functions of Hainan camellia oil. Totally, 350 volatile metabolites were detected, and heterocyclic compounds were revealed as the main contributors of strong aroma. Potential indicators for monitoring the stir-fry degree were established. Eight key aroma volatile metabolites were identified, including three new ones (1-octen-3-one, 2,3-butanedione, and vanillin). Lipids degradation and the Millard reaction are probably the main pathways for aroma generation. Over-stir-fry treatment diminished the contents of some important volatile metabolites but increased the risk of arising burnt odor. Our work offered insights into the effects of the stir-fry process and over-stir-fry treatment on Hainan camellia oil, which is meaningful for improving the hot-pressing technique.

## Highlights

• A total of 350 volatile metabolites were identified in Hainan camellia oil samples.
• The stir-fry process changed the volatile metabolite profiles of Hainan camellia oil.
• Over-stir-fry treatment diminished some important volatile metabolite contents.
• Potential indicators to monitor the stir-fry degree of making Hainan camellia oil.
• Lipid degradation and Millard reaction dominated in volatile aroma generation.

## 1 Introduction

Oil-tea camellia, endemic in China, is a common name for the woody shrub of the genus Camellia in the Theaceae family that has high seed oil content (Cao et al., 2016; Lin et al., 2022; Xia et al., 2023). In the genus Camellia, the other two well-known species are *Camellia sinensis*, whose leaves are used to produce tea, and *C. japonica*, an important ornamental plant (Cao et al., 2016; Zhang et al., 2021). For oil-tea camellia, *C. oleifera*, *C. drupifera*, *C. meiocarpa*, and *C. osmantha* are the four primarily cultivated species in the hills and mountainous areas of southern China (Chen et al., 2022; Wang et al., 2022). The primary product of oil-tea camellia is camellia oil obtained from its mature kernels. Camellia oil, a high-quality healthy edible oil recommended by the Food and Agricultural Organization, has been consumed in China for over 2300 years (Dou et al., 2024; Xia et al., 2023). Due to the similar fatty acid compositions and physiochemical properties to olive oil, camellia oil is called “oriental olive oil” (Dou et al., 2024; He et al., 2021). Former studies showed camellia oil is rich in unsaturated fatty acids (mainly oleic acid and linoleic acid, about 90%) and numerous human health-promoting active ingredients (e.g., squalene, flavonoids, tea-saponin, phytosterols, vitamins, and carotene) (Deng et al., 2024; Dou et al., 2024; Xia et al., 2023). Beneficial effects on human health of long-term consumption of camellia oil include relieving cardiovascular and cerebrovascular diseases, lowering blood cholesterol, protecting the liver, antioxidant, anti-inflammatory, etc. (Dou et al., 2024; Xia et al., 2023). Owing to its dietary and therapeutic effects, camellia oil in the market usually has a higher price than other edible oils and has become increasingly favored by consumers (Deng et al., 2024; Dou et al., 2024).

The quality and flavor of camellia oil are not only affected by various geographical and environmental factors, such as climate, soil, and water (Deng et al., 2024; Dou et al., 2024; Wang et al., 2022), but also by camellia species and process techniques. Camellia oils that were made from *C. oleifera*, *C. drupifera*, *C. meiocarpa*, and *C. osmantha* have different nutritional values, and they were welcomed in distinct provinces or regions in China (Chen et al., 2022; Deng et al., 2024; Fang et al., 2022). The process techniques of manufacturing camellia oil can be divided into hot-pressing and cold-pressing, with hot-pressing requiring the heating of mature kernels whereas cold-pressing requires no heating before pressing camellia oil. Hainan camellia oil, produced from mature kernels of local *C. drupifera* in Hainan Island using the hot-pressing technique, is well known for its outstanding quality and commercial value. Generally, Hainan camellia oil characteristics with stronger aroma, large consistency, mellow taste, slightly sweet taste, and a higher price (Deng et al., 2024; Xia et al., 2023). Besides the unique tropical climate, excellent water, and soil conditions on Hainan Island, the outstanding quality of Hainan camellia oil is strongly affected by the special traditional stir-fry hot-pressing technique, which requires stir-fry mature kernel of *C. drupifera* to the final temperature at 150 °C. However, the volatile metabolite compositions of Hainan camellia oil, the dynamic changes of volatile metabolites during the traditional stir-fry process of making Hainan camellia oil, and the negative effects of the over-stir-fry treatment in Hainan camellia oil remain mysterious.

Gas chromatography-mass spectrometry (GC–MS) was usually performed to analyze volatile metabolites in camellia oils made from mature kernels of *C. oleifera* and *C. osmantha* (Fang et al., 2022; He et al., 2021; Jia et al., 2021; Jia et al., 2024; Wang et al., 2022). However, the identification and quantification accuracy of GC–MS analysis is affected by the co-elution problems (Perestrelo et al., 2011; Xue et al., 2022). The comprehensive two-dimensional gas chromatography coupled to time-of-flight mass spectrometry (GC × GC-TOFMS), which employs two distinct stationary phase capillary columns for a single analysis, has overcome the co-elution problems of GC–MS and displays the advantages of high sensitivity, high resolution, large peak capacity, and qualitative accuracy (Xue et al., 2022). Taking advantage of GC × GC-TOFMS, it was selected to detect the volatile metabolite compositions in Hainan camellia oil samples (HCOSs) that were prepared at different stir-fry temperatures, such as at room temperature (RT), 90 °C, 105 °C, 115 °C, 150 °C, and 170 °C (over-stir-fry treatment). The network pharmacology approach based on system biology and polypharmacology, which is usually achieved using bioinformatics methods by searching public databases or comparing with available data from earlier researchers, has proved to be an effective method in measuring the regulatory effect of drugs or bioactive compounds on the biomolecular network from a systematic and holistic perspective (Hopkins, 2008; Lazzara et al., 2024; Wang et al., 2021; Zhang et al., 2013). Moreover, the network pharmacology approach has been demonstrated to be a useful tool for evaluating the human health-promoting function of metabolites in the mature kernel of *C. drupifera* (Xia et al., 2023).

In this work, the volatile metabolites of the HCOSs were analyzed using GC × GC-TOFMS, and the active ingredients and active pharmacology ingredients belonging to Traditional Chinese Medicine (TCM) in the volatile metabolites of the HCOSs were annotated using the network pharmacology approach to assess their human health-promoting function. Multivariate statistical analyses were employed to study the volatile metabolite profiles and differential volatile metabolites of the HCOSs. The relative odor activity values (ROAVs) of volatile metabolites in the HCOSs were chosen to screen the key aroma volatile metabolites. This work provides insights into the volatile metabolite compositions of Hainan camellia oil, the dynamic changes in volatile metabolites during the traditional stir-fry process of making Hainan camellia oil, the negative effects of the over-stir-fry treatment, and key aroma volatile metabolites in the HCOSs. Our findings are not only helpful in understanding why the optimized stir-fry temperature of making Hainan camellia oil is 150 °C but also offer theoretical references to improve the hot-pressing technique of making Hainan camellia oil.

## 2 Material and methods

### 2.1 Plant materials and preparation of the HCOSs

**Fig. 1:**
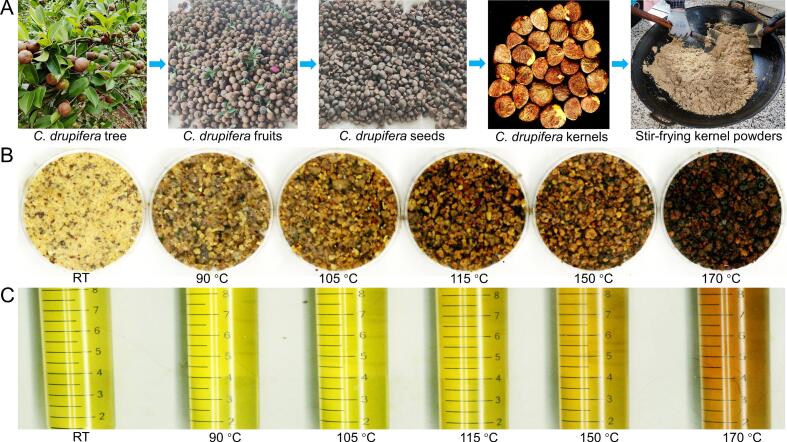
Preparation of Hainan camellia oil samples (HCOSs). (A) Main steps before hot-pressing to manufacture HCOSs; Illustration photographs of the stir-fry kernel powders of *C. drupifera* at different final temperatures (B), and their corresponding HCOSs (C).

Ripe and healthy fresh fruits from 10 years old *C. drupifera* trees were harvested in Qionghai City, Hainan Province, China, on November 25, 2022. After peeling, the resulting mature seeds were dried naturally in the sun until their moisture content was between 7% and 8%, followed by dehulled. These dehulled mature kernels were crushed by a grinder (9fz-28, Changding, Jinhua, Zhejiang, China) to generate kernel powders with a particle size of 40 mesh (Fig. 1A). In total, 12 kg of kernel powders were weighed and further submitted to a stir-fry process by experienced workers. Before the stir-fry process, 2 kg of kernel powders were weighed to prepare a control sample at RT, with the rest 10 kg of kernel powders submitted to the stir-fry process. During the stir-fry process, the temperature changes were monitored using a Deli infrared thermometer (DL333380, Ningbo, Zhejiang, China). Based on the color and scent changes of the kernel powders, each 2 kg of the stir-fry samples at 90 °C, 105 °C, 115 °C, 150 °C, and 170 °C were independently obtained, with that of 170 °C as an over-stir-fry treatment (Fig. 1B). These six samples of kernel powders were separately pressed using a fully automatic hydraulic oil press machine (280D, Wangquan, Linyi, Shangdong, China) to obtain the HCOSs. Before and after each press, the fully automatic hydraulic oil press machine was thoroughly cleaned using high-pressure air blows and cleaning papers. According to their sampled temperatures, these six HCOSs were designated as COSRT, COS90, COS105, COS115, COS150, and COS170, respectively (Fig. 1C). After being centrifuged at 6000*g*/min for 10 min, these six HCOSs were individually submitted to volatile metabolite detections.

### 2.2 Chemical reagents

Chromatographic purity ethanol (99.8%) was purchased from Aladdin (Shanghai, China). *n*-alkane (C_7_-C_30_, analytical standard) for linear retention index (RI) determination, 1-Hexan-d13-ol (internal standard), and *n*-hexane (analytical standard) were obtained from Sigma-Aldrich (Shanghai) Trading Co. Ltd. (Shanghai, China). Ultrapure water was purified by a Milli-Q purification system (Millipore, Bedford, MA, USA).

### 2.3 Sensory evaluation

The sensory evaluation of the HCOSs was conducted in a suitable laboratory environment with a constant temperature of 25 °C referring to previous studies with some modifications (Cao et al., 2016; He et al., 2021; Jia et al., 2024). Briefly, seven well-trained panelists (four males and three females, aged 25–35) familiar with the flavor of Hainan camellia oil were selected for sensory evaluation, with each 5.0 g HCOS placed into a 10 mL glass vial. Eight main odor descriptors, including woody, green, sweet, fruity, fatty, roasted, nutty, and caramellic, were recorded on a 10-point scale ranging from 0 (not perceivable) to 10 (strongly perceivable). The average sensory values of the seven panelists were plotted on a radar chart.

### 2.4 Extraction of volatile metabolites in the HCOSs using headspace solid phase microextraction (HS-SPME)

The volatile metabolites in the HCOSs were extracted following previous documents with minor modifications (Cao et al., 2016; He et al., 2021; Jia et al., 2021). Initially, 1 mL of each Hainan COS was precisely measured and transferred into a 20 mL headspace vial (Agilent). Then, 10 μL internal standard solution (1 mg/L) was added. Following the headspace vial was immediately sealed with a PTFE/silicone cap (Agilent) the Supelco 50/30 μm DVB/CAR/PDMS fiber (57329-U, Bellefonte, PA, USA) was introduced into the headspace vial to absorb the volatile metabolites. The incubation was performed at a consistent temperature of 50 °C for 30 min. After absorption, the fiber was inserted into the GC × GC-TOFMS injector to desorb the analytes at 250 °C for 5 min.

### 2.5 GC × GC-TOFMS analyses of the HCOSs

The volatile metabolites in the HCOSs were investigated using a LECO Pegasus BT 4D GC × GC-TOFMS instrument (LECO Corporation, St. Joseph, MI, USA), consisting of an Agilent 8890 Gas Chromatograph system (Agilent Technologies Inc., Palo Alto, CA, USA) equipped with a split/splitless injector and a dual stage cryogenic modulator (LECO) coupled with TOFMS detector (LECO). DB-Heavy Wax (30 m × 250 μm × 0.5 μm; Agilent) and Rxi-5Sil MS (2.0 m × 150 μm × 1.5 μm; Resteck, Bellefonte, PA, USA) columns were employed as the first-dimensional (1D) and second-dimensional (2D) separation, respectively. The GC × GC-TOFMS conditions were optimized according to some previous documents (Yang et al., 2022; Ruan et al., 2023). In brief, high-quality helium (>99.999%) was used as the carrier gas at a constant flow velocity of 1.0 mL/min. The temperature of the GC injector was kept at 250 °C. The oven temperature of the 1D column was programmed as follows: initial temperature, 40 °C (hold for 2 min); increasing at 5 °C/min to 250 °C (hold for 5 min). The oven temperature of the 2D column was operated at 5 °C higher than that of the 1D column, while always 15 °C lower than that of the modulator, with a 4.0 s modulation period. The transfer line and TOF MS ion source temperatures were set at 250 °C. The acquisition frequency was 200 spectra/s. The mass spectrometer was operated in the electron ionization (EI) mode, with ionization energy at 70 eV, mass scan ranged from *m*/*z* 35 to 550, and detector voltage at 2020 V.

### 2.6 Qualitative and quantitative determination of the volatile metabolites in the HCOSs

The qualitative and quantitative determinations of the volatile metabolites in the HCOSs were performed following previous documents with minor modifications (Wang et al., 2023; Xue et al., 2022; Yang et al., 2022). In short, the data obtained by GC × GC-TOFMS were processed by ChromaTOF software (LECO) and annotated by the mass spectrometry library of the National Institute of Standards and Technology (NIST2020) and self-built database (Panomix Biomedical Tech Co. Ltd., Suzhou, Jiangsu, China). Based on the minimum similarity match set at 700, the RI values calculated by the *n*-alkane standards (C_7_-C_30_) were compared with those in the NIST Chemistry WebBook (https://webbook.nist.gov/chemistry/). The semi-quantitative method with 1-Hexan-d13-ol as an internal standard was employed to do relative quantification of volatile metabolites in the HCOSs. The calculation equation is as follows: (1) the relative concentration of volatile metabolites = (volatile compounds peak area/internal standard peak area) × internal standard concentration.

### 2.7 Evaluation of key volatile metabolites in the HCOSs

The relative odor activity value (ROAV) method was conducted to evaluate the key volatile metabolites in the HCOSs (Fang et al., 2022; Xue et al., 2022). The calculation formula of ROAV is as follows:

(2) $$\text{ROAV}=\frac{C_{i}}{T_{i}}\times \frac{T_{\max}}{C_{\max}}\times 100$$

Where C_i_ and T_i_ indicate the relative content of each volatile metabolite and the corresponding odor threshold, respectively. T_max_ and C_max_ point to the relative content and corresponding odor of the metabolites with the maximum C_i_/T_i_ value. ROAV value ranges from 0 to 100. Generally, the higher ROAV value means the higher odor contrition of the volatile metabolites to the overall flavor. These volatile metabolites with ROAV ≥1 were considered key odor volatile metabolites in the HCOSs. Odor thresholds of volatile metabolites in the HCOSs were obtained by survey literature (He et al., 2021; Jia et al., 2019; Jia et al., 2021; Jia et al., 2024; Zhu et al., 2020).

### 2.8 Annotation of active volatile metabolites with human health-promoting functions in the HCOSs

According to our previous work (Xia et al., 2023), the active ingredients and active pharmaceutical ingredients belonging to TCM in volatile metabolites of the HCOSs were annotated using the network pharmacology approach.

### 2.9 Statistical analysis

Multivariate statistical analyses, including principal component analysis (PCA) and orthogonal partial least-squares discriminant analysis (OPLS-DA), were conducted using *Z*-score standardized metabolic data of COSs in R software (version 4.3.2). In OPLS-DA, Z-score standardized metabolic data were further log_2_-transformed, differential metabolites selection criteria in the pairwise comparisons were set to *P* value <0.05 and the threshold variable importance in projection (VIP) value >1, and permutation tests were validated with 200 permutations.

The relative contents of volatile metabolites in the HCOSs were shown as means (± SD) and statistically analyzed using Duncan's multiple comparison test by SAS software (version 9.4). The radar chart of the sensory evaluation was drawn by OriginPro 2021 software. Venn diagram was generated by the UpSetR package.

## 3 Results and discussion

### 3.1 Sensory evaluation of the HCOSs

**Fig. 2:**
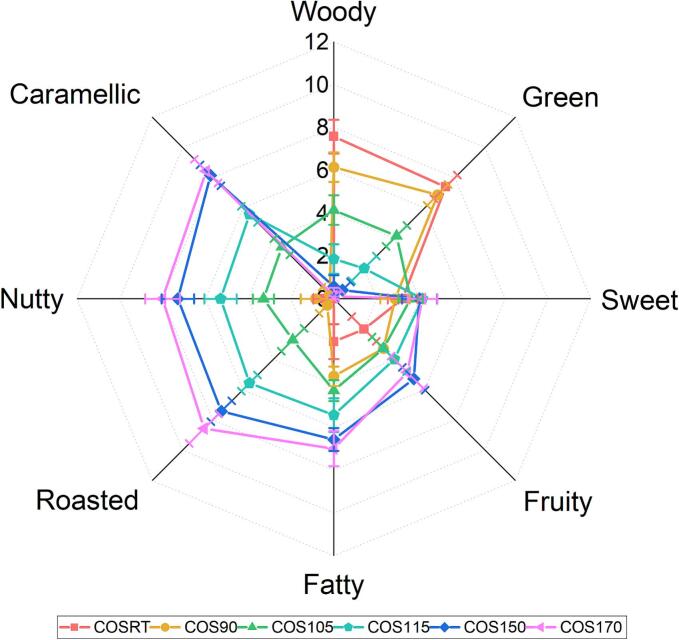
Sensory evaluation of these six HCOSs.

Based on the flavor descriptions of camellia oil made from *C. oleifera* mature kernels (He et al., 2021; Jia et al., 2024) and the scent characteristics of Hainan camellia oil (Deng et al., 2024; Xia et al., 2023), eight characteristic odor descriptors, consisting of woody, green, sweet, fruity, fatty, roasted, nutty, and caramellic, were selected to evaluate the HCOSs. As shown in Fig. S1A, the PCA distribution diagram of the sensory scores indicates the volatile metabolite profiles of COSRT, COS90, COS105, COS115, COS150, and COS170 were different. However, the volatile metabolite profiles of COSRT and COS150 were relatively close to that of COS90 and COS150, respectively. Further radar chart (Fig. 2) and multiple comparisons of the sensory scores of these eight descriptors (Fig. S1B) reveal the green and woody scores were highest in COSRT and COS90. In contrast, roasted, caramellic, nutty, fatty, and fruity scores were highest in COS150 and COS170. In COS105 and COS115, the sensory scores of all eight odor descriptors were intermediate, with green and woody sensory scores higher in COS105, and caramellic, nutty, roasted, and fatty scores higher in COS115 (Fig. 2; Fig. S1B). Notably, the sweet scores among these six HCOSs were close (Fig. 2; Fig. S1B), suggesting the sweet odor of the HCOSs changed slightly during the stir-fry process. Compared the COS150 to the over-stir-fry COS170 (Fig. 1B; Fig. 2; Fig. S1B), we found sensory scores of seven odor descriptors (woody, green, sweet, fruity, fatty, nutty, and caramellic) were statistically not significantly different, and that of roasted was significantly higher in COS170 than in COS150, indicating the over-stir-fry treatment cannot improve the odor of Hainan camellia oil but increased the risk of arising burnt odor. Collectively, these results demonstrated the green and woody odors gradually decreased, and the roasted, caramellic, nutty, fatty, and fruity odors gradually increased during the stir-fry process, and the 150 °C should be optimized stir-fry temperature for making Hainan camellia oil.

During the stir-fry process, the odors of the HCOSs were changed from “fresh fragrance” (characterized by green and woody) to “strong fragrance” (described by roasted, caramellic, nutty, fatty, and fruity) with the stir-fry temperature raised from RT to 170 °C, which is consistent with earlier reported heating processes of making camellia oil from *C. oleifera* (He et al., 2021; Jia et al., 2024). The odor changes of the HCOSs are probably caused by the loss of some alcohols and esters that contribute to “fresh fragrance” and the gain of some heterocycles and aldehydes that respond to “strong fragrance” (He et al., 2021). The reason why over-stir-fry treatment cannot significantly change the odor of Hainan camellia oil might be due to the Millard reaction and the Strecker degradation being sufficient at 150 °C during the stir-fry process.

### 3.2 Investigation of the volatile metabolite compositions and their dynamic changes in the HCOSs using the HS-SPME/GC × GC-TOFMS

**Fig. 3:**
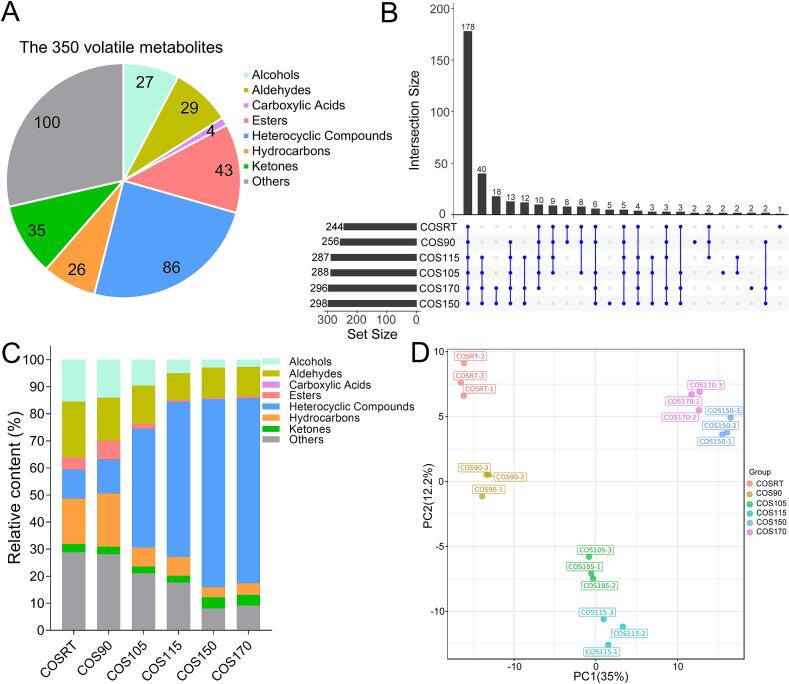
The profiles of volatile metabolites in these six HCOSs were different. (A) Classification of the 350 volatile metabolites identified in the HCOSs; (B) Venn diagram of the volatile metabolites in the HCOSs; (C) The relative contents of the eight categories of volatile metabolites in the HCOSs; (D) The result of principal component analysis using the 350 volatile metabolites.

To understand the volatile metabolite bases of the odor changes during the stir-fry process, the volatile metabolite compositions of the HCOSs were investigated using HS-SPME/GC × GC-TOFMS, and the dynamic changes of volatile metabolites of the HCOSs were analyzed. A total of 350 volatile metabolites were identified in the HCOSs, involving 27 alcohols, 29 aldehydes, 4 carboxylic acids, 43 esters, 86 heterocyclic compounds, 26 hydrocarbons, 35 ketones, and 100 others (Fig. 3A). The number of volatile metabolites detected in COSRT, COS90, COS105, COS115, COS150, and COS170 was 244, 256, 288, 287, 298, and 296, respectively, with 178 co-detected volatile metabolites (Fig. 3B). Moreover, special volatile metabolites that only detected in COSRT, COS90, COS105, COS150, or COS170 were also revealed (Fig. 3B; Table S1). These special volatile metabolites can be explored as biomarkers to distinguish the stir-fry degree of Hainan camellia oil. The five special volatile metabolites only detected in COS150, including 1,4-cyclohexanedione, ethyl (E)-2-crotonate, hexylbenzene, cyclopentanecarboxaldehyde, and 2,4-dimethyl-1,3-oxazole, could be treated as indicators for the completion of the stir-fry process of Hainan camellia oil. Meanwhile, the two special volatile metabolites only detected in COS170 (diethyl butanedioate, and 2-acetyl-5-methylfuran) might be explored as indicators for the over-stir-fry of Hainan camellia oil.

Relative contents of the eight categories identified in the HCOSs exhibited different change trends during the stir-fry process (Fig. 3C). When the stir-fry temperatures ranged from RT to 150 °C, the relative contents of alcohols and others exhibited a decreasing trend. At the same time, that of heterocyclic compounds was gradually increased. The relative contents of aldehydes, esters, and hydrocarbons were higher in COSRT and COS90, but lower in COS150 and COS170. On the contrary, carboxylic acids were not detected in COSRT and COS90 but were detected in COS150 and COS170. The relative contents of ketones were similar among all COSs, ranging from 2.57% (in COS115) to 4.28% (in COS150), with an average of 3.22%. Notably, the relative contents of heterocyclic compounds in COS150 and COS170 were 69.14% and 68.28%, respectively, indicating heterocyclic compounds might be the main contributor to the strong aroma of COS150 and COS170. Consistent with Fig. 3C, the PCA result also revealed the volatile metabolite profiles were different among these six HCOSs (Fig. 3D). Due to being clustered relatively close, the volatile metabolite profiles of COS105 and COS150 were similar to those of COS115 and COS170, respectively. Summarized, these results confirmed the volatile metabolite profiles of these six HCOSs were modified during the stir-fry process and suggested alcohols and esters might respond to “fresh fragrance” in COSRT and COS90 whereas heterocyclic compounds contribute to “strong fragrance” in COS150 and COS170, which is in agreement with former reports on camellia oil from *C. oleifera* (He et al., 2021; Jia et al., 2019; Zeng et al., 2022). Notably, the heterocyclic compounds in COS150 and COS170 might have originated from the Millard reaction and the Streck degradation (He et al., 2021; Ho et al., 2015; Parker, 2015; Schwab et al., 2008).

### 3.3 The stir-fry process modified the human heal-promoting function of the HCOSs

To assess if the human health-promoting functions of the HCOSs were modified by the stir-fry process, the 350 volatile metabolites identified in this work were annotated using the network pharmacology approach (Xia et al., 2023). In total, 200 volatile metabolites (57.14%), consisting of 100 ones related to at least one target protein and disease, 15 ones associated with at least one target protein, and 85 ones linked to no target protein and disease, were uncovered to be the chemical composition belonging to TCM (Table S2). Unfortunately, none of them can satisfy the key active ingredients select criteria of OB ≥ 5% and DL ≥ 0.14 (Xia et al., 2023), with all DL values of them <0.14. Fortunately, four volatile metabolites, including linalool, methyl isovalerate, methyl eugenol, and vanillin, were annotated as active pharmaceutical ingredients belonging to TCM (Table S2). All four active pharmaceutical ingredients were annotated anti-Alzheimer's disease, vanillin was annotated anti-cancer, methyl eugenol and vanillin were co-annotated anti-myocardial infarction, anti-inflammation, anti-arthritis, and anti-stroke (Ru et al., 2014). No volatile metabolite of the HCOSs was involved in anti-cardiovascular diseases, indicating the anti-cardiovascular disease function of Hainan camellia oil is probably concentrated in non-volatile metabolites (Deng et al., 2024; Dou et al., 2024; Lin et al., 2022).

**Fig. 4:**
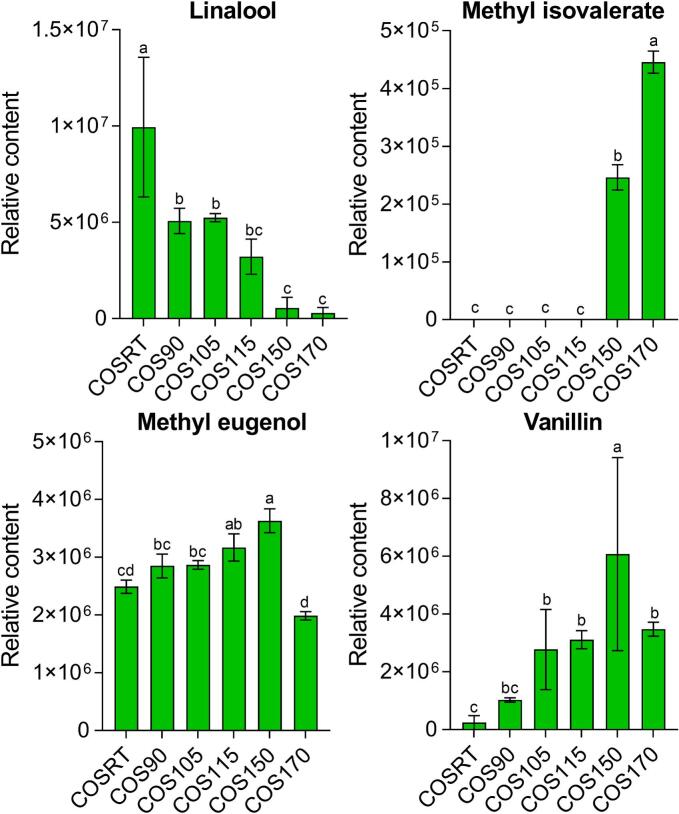
The relative contents of the four annotated active pharmaceutical ingredients belonging to traditional Chinese medicine (TCM) in the HCOSs.

Among the four annotated active pharmaceutical ingredients belonging to TCM, the relative contents of methyl isovalerate, methyl eugenol, and vanillin were highest in COS150, with linalool that annotated only anti-Alzheimer's disease relatively lower in COS150 (Fig. 4). For methyl isovalerate, the other volatile metabolite that annotated only anti-Alzheimer's disease, it was barely detected in COSRT, COS90, COS105, and COS115 but showed relatively higher content in COS150 and COS170. For methyl eugenol and vanillin, which annotated involved in anti-multiple diseases, the relative contents were highest in COS150 and exhibited an increasing trend from RT to 150 °C, with a significantly decreased relative content in the over-stir-fry COS170 (Fig. 4). These results suggest the human health-promoting function of Hainan camellia oil probably enhanced during the stir-fry process to some extent but probably diminished by the over-stir-fry treatment.

### 3.4 OPLS-DA comparison reveals differential volatile metabolites during the stir-fry process

**Fig. 5:**
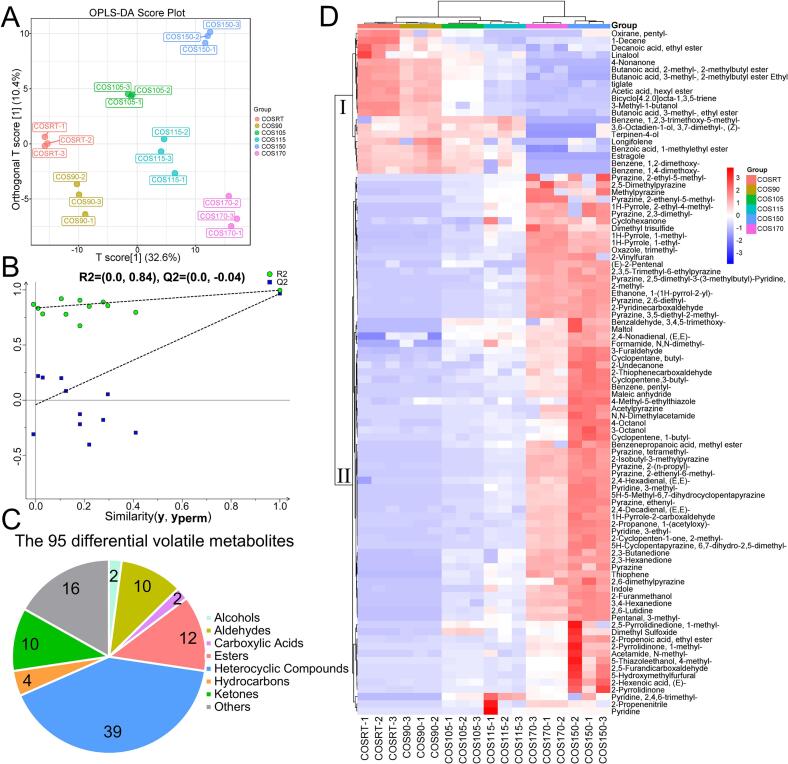
Multivariate statistical analysis on the volatile metabolites in the HCOSs. (A) The score plot of OPLS-DA; (B) The verification plot by 200 permutation tests; (C) Classification of the 95 differential volatile metabolites; (D) The cluster heatmap of the 95 differential volatile metabolites.

To identify the differential volatile metabolites that respond to the stir-fry process, the OPLS-DA comparison of all these six HCOSs (COSRT vs. COS90 vs. COS105 vs. COS115 vs. COS150 vs. COS170) was conducted (Fig. 5). The parameters of the OPLS-DA model displayed robust interpretive and predictive abilities, with R^2^Y = 0.997 and Q^2^ = 0.967. As shown in Fig. 5A, the OPLS-DA score plot separated these six HCOSs, indicating the volatile fingerprints underwent continuous dynamic changes during the stir-fry process. The rationality and feasibility of the OPLS-DA model were further demonstrated by the 200 permutation tests, with R^2^ = 0.84 and Q^2^ = -0.04 (Fig. 5B). Under the screen criterion of VIP values >1 and *p*-value <0.05, the suitable OPLS-DA comparison revealed 95 differential volatile metabolites, including 2 alcohols, 10 aldehydes, 2 carboxylic acids, 12 esters, 39 heterocyclic compounds, 4 hydrocarbons, 10 ketones, and 16 others, during the stir-fry process (Fig. 5C and D). Thirty-eight of these 95 differential volatile metabolites were annotated active ingredients in TCM (Table S2), which further confirmed the human health-promoting function of Hainan camellia oil was modified by the stir-fry process. These 95 differential volatile metabolites can be broadly categorized into two groups (Fig. 5D). The 20 volatile metabolites included in group I displayed a decreasing trend with the temperature increase during the stir-fry process. Meanwhile, the remaining 75 volatile metabolites contained in group II exhibited a contrary trend from RT to 150 °C, implying they were generated during the stir-fry process. Interestingly, 3-methyl-1-butanol in group I was also documented to have the highest concentration in the untreated camellia oil from *C. oleifera* (He et al., 2021). Five volatile metabolites in group II, including 2,6-dimethylpyrazine, 2,5-dimethylpyrazine, 2-furanmethanol, methylpyrazine, and (E)-2-pentenal, were also reported to have higher concentrations in microwave pretreatment camellia oil from *C. oleifera* (He et al., 2021). It is worth noting that the relative contents of most volatile metabolites in group II were higher in COS150 than in COS170, indicating the over-stir-fry treatment could cause the content reduction of differential volatile metabolites in Hainan camellia oil. Collectively, the differential volatile metabolites uncovered by OPLS-DA comparison and their change trends of relative contents illustrated by the heatmap were reasonable and reliable.

### 3.5 Screen the key aroma volatile metabolites in the HCOSs based on ROAVs

Based on documented (Fang et al., 2022; He et al., 2021; Jia et al., 2024; Xue et al., 2022), the contribution of a volatile compound to the overall aroma profile depends on its concentration and odor threshold. In this work, the ROAVs calculated from the relative content of each volatile metabolite and the corresponding odor threshold were employed to screen the key aroma volatile metabolites in these six HCOSs. According to the selection criteria of ROAV ≥1, eight key aroma volatile metabolites were obtained in the HCOSs. Three key aroma volatile metabolites (Table 1), including 1-octen-3-one, 2,3-butanedione, and vanillin, were first identified in Hainan camellia oil. The other five key aroma volatile metabolites (Table 1), consisting of (E)-2-nonenal, (E)-2-octenal, 2,3,5-trimethylpyrazine, 2-pentylfuran, and heptanal, were also previously identified in camellia oils made from *C. oleifera*, and *C. osmantha* (Fang et al., 2022; He et al., 2021; Ho et al., 2015; Jia et al., 2024). Compared to the other seven key aroma volatile metabolites (Table 1), the ROVAs of (E)-2-nonenal were the highest in all these six HCOSs, suggesting (E)-2-nonenal might be an important fundamental key aroma volatile metabolite in Hainan camellia oil. Moreover, the ROAVs of (E)-2-nonenal in COS150 and COS170 were 100 and 97.94, respectively. For (E)-2-octenal, 2,3-butanedione, and vanillin, their ROAVs were highest in COS150, indicating they were crucial aroma contributors in Hainan camellia oil.

**Table 1:** Eight key aroma volatile metabolites identified in the HCOSs.

Volatile metabolites	Odor threshold (mg/kg)	Odor descriptions	ROAVs					
			COSRT	COS90	COS105	COS115	COS150	COS170
(E)-2-Nonenal	0.00008	Fatty, cucumber	36.14	33.97	42.40	49.70	100.00	97.94
(E)-2-Octenal	0.003	Nuts, green, fatty	4.36	3.17	3.12	2.90	7.00	6.09
1-Octen-3-one	0.000005	Mushroom-like	1.44	0.33	0.69	0.00	2.96	4.21
2,3,5-Trimethylpyrazine	0.4	Roasted nuts, cocoa, peanuts	0.53	0.66	4.95	9.30	9.06	9.79
2,3-Butanedione	0.002	Pleasant, buttery	0.00	0.40	5.03	5.36	20.14	16.80
2-Pentylfuran	0.006	Green beans, vegetable	19.23	13.31	21.33	21.21	10.21	24.55
Heptanal	0.003	Citrus, fatty, rancid	23.46	12.78	10.06	8.26	20.31	20.91
Vanillin	0.02	Vanilla, caramel, sweet	1.09	4.62	12.50	14.02	27.37	15.67

The aroma compounds in the heating process are mainly generated from four pathways, including the Millard reaction pathway, lipids as precursors, glycosides as precursors, and carotenoids as precursors (Chen et al., 2019; Ho et al., 2015; Schwab et al., 2008; Wang et al., 2024; Yang et al., 2013). Among these eight key aroma volatile metabolites in the HCOSs, the generate pathways of heptanal, 2-pentylfuran, 1-octen-3-one, 2,3-butanedione, and 2,3,5-trimethylpyrazine have been reported in the heating process of tea (Ho et al., 2015; Wang et al., 2024). Heptanal, 2-pentylfuran, 1-octen-3-one, and 2,3-butanedione are categorized as fatty-acid derived volatiles (FADVs), and their precursors are lipids (Ho et al., 2015; Wang et al., 2024). Heptanal is mainly converted from 8-hydroperoxypalmitoleic acid, an oxidization product of palmitoleic acid catalyzed by lipoxygenase (LOX) (Caipo et al., 2021; Wang et al., 2024). 2-pentylfuran is an oxidation product of linoleic acid catalyzed by amino acids during the thermal processing of lipid-rich foods (Wang et al., 2024). 1-octen-3-one, subsequently converting to 1-octen-3-ol under the catalysis of alcohol dehydrogenase (ADH), is also derived from linoleic acid (Wang et al., 2024). 2,3,5-trimethylpyrazine is a product of the reaction of theanine and d-glucose or other monosaccharides in the Millard reaction pathway (Ho et al., 2015). Since Hainan camellia oil is rich in lipids, amino acids, and monosaccharides, especially linoleic acid and palmitoleic acid, like tea, we speculate that the main formation pathways of heptanal, 2-pentylfuran, 1-octen-3-one, 2,3-butanedione, and 2,3,5-trimethylpyrazine are similar in tea and Hainan camellia oil. However, the main generate pathways of (E)-2-nonenal, (E)-2-octenal, and vanillin, the other three key aroma volatile metabolites in Hainan camellia oil, still need to be explored.

## 4 Conclusion

Sensory evaluation, HS-SPME/GC × GC-TOFMS analysis, the network pharmacology approach, and multivariate statistical analyses were performed to uncover the effects of the stir-fry process and over-stir-fry treatment on Hainan camellia oil. A total of 350 volatile metabolites were identified from the HCOSs. The traditional stir-fry process modified the odors, volatile metabolite profiles, and human health-promoting functions of the HCOSs. The over-stir-fry treatment diminished the contents of some volatile metabolites with human health-promoting function and differential volatile metabolites generated by the traditional stir-fry process, but increased the risk of arising burnt odor. Five volatile metabolites specially detected in COS150 (1,4-cyclohexanedione, ethyl (E)-2-crotonate, hexylbenzene, cyclopentanecarboxaldehyde, and 2,4-dimethyl-1,3-oxazole) could be treated as indicators for the completion of the stir-fry process of Hainan camellia oil. Two volatile metabolites only identified in the over-stir-fry treatment sample COS170 (diethyl butanedioate, and 2-acetyl-5-methylfuran) have the potential to be explored as indicators to monitor the over-stir-fry of Hainan camellia oil. The 95 differential volatile metabolites revealed by the OPLS-DA comparison can be generally categorized into two groups. Eight volatile metabolites with ROAV ≥1 ((E)-2-nonal, (E)-2-octenal, 1-octen-3-one, 2,3,5-trimethylpyrazine, 2,3-butanedione, 2-pentylfuran, heptanal, and vanillin) were considered key aroma volatile metabolites in Hainan camellia oil. Furthermore, the lipids degradation and Millard reaction probably served as the dominant pathways for aroma generation. Our work offered comprehensive information on the volatile metabolite compositions, the effects of stir-fry, and the negative effects of over-stir-fry treatment on the volatile metabolite profiles in the HCOSs. These potential indicators are helpful in monitoring the stir-fry degree of Hainan camellia oil, which is meaningful to improve the quality of Hainan camellia oil.

## Ethical statement

Informed consent of all participants was obtained before participating in the sensory evaluation of Hainan camellia oil samples. Appropriate protocols for protecting the rights and privacy of all participants were utilized during the execution of the sensory evaluation of Hainan camellia oil. All tested Hainan camellia oil samples were safe for consumption. We certify that the sensory evaluation panel research of Hainan camellia oil samples, approved by the Institute of Tropical Horticulture Research, Hainan Academy of Agricultural Sciences, was performed in strict accordance with the current laws of China.

## CRediT authorship contribution statement

**Tengfei Xia:** Writing – review & editing, Writing – original draft, Investigation, Funding acquisition, Conceptualization. **Zijun Xiong:** Writing – original draft, Methodology, Investigation, Formal analysis. **Chunmei Wang:** Investigation. **Xiuxiu Sun:** Investigation. **Yeguang Chen:** Investigation. **Jiali Chen:** Investigation. **Huasha Qi:** Investigation. **Heng Liang:** Investigation. **Lang Zhang:** Investigation. **Daojun Zheng:** Writing – review & editing, Supervision, Funding acquisition, Conceptualization.

## Declaration of competing interest

The authors declare that they have no known competing financial interests or personal relationships that could have appeared to influence the work reported in this paper.

## Data Availability

The data that has been used is confidential.

## Appendix A Supplementary data

Supplementary material Image 1
