# Supplementary material for: Comprehensive analysis of the effects of the traditional stir-fry process on the dynamic changes of volatile metabolites in Hainan camellia oil
Source: Food Chem X. 2024 Aug 17;23:101747. doi: 10.1016/j.fochx.2024.101747 (PMC11388339; doi:10.1016/j.fochx.2024.101747)
Supplement: Supplementary file 1 — Supplementary material [file mmc1.docx]

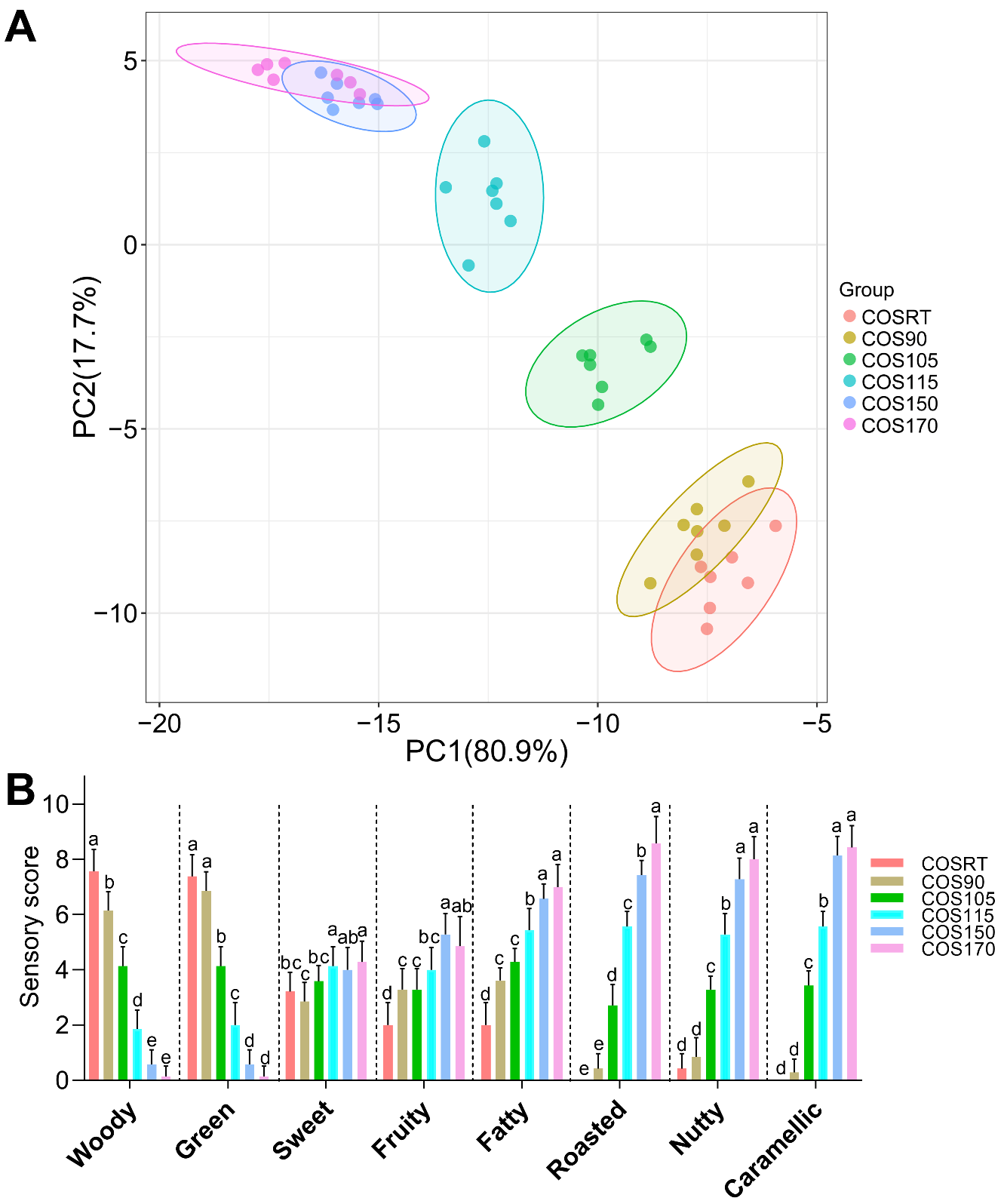


**Fig. S1.** The odor profiles of the HCOSs were different. (A) PCA plot of the HCOSs using the sensory scores of the eight odor descriptors; (B) Duncan’s multiple comparison tests of the sensory scores of the eight odor descriptors. Odor descriptors were separated with the dashed line. Different letters of each odor descriptor indicate significantly different (P <0.05).

**Table S1** The special volatile metabolites that were only detected in one kind of Hainan camellia oil sample

| **Volatile metabolite name** | **CAS** | **Chemical formula** | **Special detected in HCOSs** |
| --- | --- | --- | --- |
| 1,4-cyclohexanedione | 637-88-7 | C_6_H_8_O_2_ | COS150 |
| ethyl (E)-2-crotonate | 623-70-1 | C_6_H_10_O_2_ |  |
| hexylbenzene | 1077-16-3 | C_12_H_18_ |  |
| cyclopentanecarboxaldehyde | 872-53-7 | C_6_H_10_O |  |
| 2,4-dimethyl-1,3-oxazole | 7208-05-1 | C_5_H_7_NO |  |
| 1,2,3-trimethylbenzene | 526-73-8 | C_9_H_12_ | COS105 |
| 2,3-dehydro-1,8-cineole | 92760-25-3 | C_10_H_16_O |  |
| diethyl butanedioate | 123-25-1 | C_8_H_14_O_4_ | COS170 |
| 2-acetyl-5-methylfuran | 1193-79-9 | C_7_H_8_O_2_ |  |
| 3-undecanone | 2216-87-7 | C_11_H_22_O | COSRT |
| 1,6-dimethyl-4-(1-methylethyl)naphthalene | 483-78-3 | C_15_H_18_ | COS90 |
| selina-3,7(11)-diene | 6813-21-4 | C_15_H_24_ |  |

**Table S2** Annotated active ingredients and active pharmaceutical ingredients related to human health in Hainan camellia oil samples belonging to Traditional Chinese Medicines (TCM)

| **Volatile Metabolite Name** | **CAS** | **Related diseases numbers** | **Related target numbers** | **Oral bioavailability (%)** | **Drug-likeness** | **Anti-cancer** | **Anti-Myocardial infarction** | **Anti-cardiovascular diseases** | **Anti-inflammation** | **Anti-arthritis** | **Anti-alzheimer's** | **Anti-stroke** |
| --- | --- | --- | --- | --- | --- | --- | --- | --- | --- | --- | --- | --- |
| Linalool | 78-70-6 | 13 | 4 | 58.18 | 0.02 | - | - | - | - | - | Yes | - |
| Methyl isovalerate | 556-24-1 | 5 | 6 | 55.9 | 0.01 | - | - | - | - | - | Yes | - |
| Methyl eugenol | 93-15-2 | 91 | 25 | 73.36 | 0.04 | - | Yes | - | Yes | Yes | Yes | Yes |
| Vanillin | 121-33-5 | 48 | 11 | 52 | 0.03 | Yes | Yes | - | Yes | Yes | Yes | Yes |
| 1,2-Benzenedicarboxylic acid, bis(2-methylpropyl) ester | 84-69-5 | 46 | 14 | 49.63 | 0.13 | - | - | - | - | - | - | - |
| 1-Octanol | 111-87-5 | 0 | 1 | 21.06 | 0.01 | - | - | - | - | - | - | - |
| 2-Decen-1-ol, (E)- | 18409-18-2 | 0 | 0 | 29.92 | 0.02 | - | - | - | - | - | - | - |
| 2-Nonanone | 821-55-6 | 0 | 0 | 8.51 | 0.02 | - | - | - | - | - | - | - |
| 2-Propenoic acid, butyl ester | 141-32-2 | 0 | 0 | 57.32 | 0.01 | - | - | - | - | - | - | - |
| 5,9-Undecadien-2-one, 6,10-dimethyl-, (E)- | 3796-70-1 | 0 | 0 | 18.66 | 0.04 | - | - | - | - | - | - | - |
| 5-Hydroxymethylfurfural | 67-47-0 | 71 | 20 | 45.07 | 0.02 | - | - | - | - | - | - | - |
| Benzene, n-butyl- | 104-51-8 | 1 | 2 | 39.45 | 0.02 | - | - | - | - | - | - | - |
| Dibutyl phthalate | 84-74-2 | 97 | 22 | 64.54 | 0.13 | - | - | - | - | - | - | - |
| Indole | 120-72-9 | 5 | 6 | 34.38 | 0.03 | - | - | - | - | - | - | - |
| Pentadecane | 629-62-9 | 0 | 0 | 13.98 | 0.05 | - | - | - | - | - | - | - |
| Pyridine, 2-methyl- | 109-06-8 | 5 | 6 | 47.89 | 0.01 | - | - | - | - | - | - | - |
| Tetradecane | 629-59-4 | 12 | 1 | 15.94 | 0.04 | - | - | - | - | - | - | - |
| 1,3-Octadiene | 1002-33-1 | 1 | 2 | 34.53 | 0.01 | - | - | - | - | - | - | - |
| 1,5-Heptadiene, 2,6-dimethyl- | 6709-39-3 | 0 | 0 | 20.13 | 0.01 | - | - | - | - | - | - | - |
| 3-Methyl-1-butanol | 123-51-3 | 3 | 8 | 65.67 | 0 | - | - | - | - | - | - | - |
| 3-Methyl-1-butanol acetate | 123-92-2 | 0 | 1 | 36.71 | 0.01 | - | - | - | - | - | - | - |
| 1-Decene | 872-05-9 | 1 | 1 | 32.69 | 0.01 | - | - | - | - | - | - | - |
| 1-Dodecanol, 3,7,11-trimethyl- | 6750-34-1 | 0 | 0 | 11.87 | 0.06 | - | - | - | - | - | - | - |
| 1-Dodecene | 112-41-4 | 0 | 0 | 17.74 | 0.02 | - | - | - | - | - | - | - |
| 1-Heptanol | 111-70-6 | 0 | 0 | 17.22 | 0.01 | - | - | - | - | - | - | - |
| 1-Hexanol | 111-27-3 | 0 | 1 | 22.04 | 0.01 | - | - | - | - | - | - | - |
| 1-Hexanol, 2-ethyl- | 104-76-7 | 0 | 0 | 26.12 | 0.01 | - | - | - | - | - | - | - |
| 1H-Pyrrole, 1-ethyl- | 617-92-5 | 4 | 4 | 48.13 | 0.01 | - | - | - | - | - | - | - |
| 1H-Pyrrole, 1-methyl- | 96-54-8 | 2 | 4 | 46.53 | 0.01 | - | - | - | - | - | - | - |
| 1H-Pyrrole, 2-methyl- | 636-41-9 | 2 | 5 | 46.64 | 0.01 | - | - | - | - | - | - | - |
| 1H-Pyrrole-2-carboxaldehyde | 1003-29-8 | 2 | 5 | 41.58 | 0.01 | - | - | - | - | - | - | - |
| 1H-Pyrrole-2-carboxaldehyde, 1-methyl- | 1192-58-1 | 4 | 6 | 52.35 | 0.01 | - | - | - | - | - | - | - |
| 1-Hydroxy-2-butanone | 5077-67-8 | 2 | 10 | 63.8 | 0 | - | - | - | - | - | - | - |
| 1-Nonanol | 143-08-8 | 1 | 2 | 33.19 | 0.01 | - | - | - | - | - | - | - |
| 1-Nonene | 124-11-8 | 1 | 1 | 34.74 | 0.01 | - | - | - | - | - | - | - |
| 1-Octen-3-one | 4312-99-6 | 0 | 0 | 19.82 | 0.01 | - | - | - | - | - | - | - |
| 1-Pentanol | 71-41-0 | 1 | 4 | 76.16 | 0 | - | - | - | - | - | - | - |
| 1-Pentanol, 3-methyl- | 589-35-5 | 1 | 2 | 36.44 | 0.01 | - | - | - | - | - | - | - |
| 1-Pentanol, 4-methyl- | 626-89-1 | 0 | 0 | 53.21 | 0.01 | - | - | - | - | - | - | - |
| 1-Penten-3-ol | 616-25-1 | 10 | 10 | 72.18 | 0 | - | - | - | - | - | - | - |
| 1-Penten-3-one | 1629-58-9 | 0 | 5 | 69.46 | 0 | - | - | - | - | - | - | - |
| 1-Undecene | 821-95-4 | 0 | 0 | 17.99 | 0.02 | - | - | - | - | - | - | - |
| 2-(4-Methoxyphenyl)ethanol | 702-23-8 | 0 | 0 | 19.21 | 0.03 | - | - | - | - | - | - | - |
| 2,3-Butanedione | 431-03-8 | 74 | 71 | 46.29 | 0.01 | - | - | - | - | - | - | - |
| 2,3-Pentanedione | 600-14-6 | 0 | 0 | 23.44 | 0.01 | - | - | - | - | - | - | - |
| 2,4-Decadienal, (E,E)- | 25152-84-5 | 8 | 8 | 51.03 | 0.02 | - | - | - | - | - | - | - |
| 2,4-Heptadienal, (E,E)- | 4313-03-5 | 0 | 0 | 19.14 | 0.01 | - | - | - | - | - | - | - |
| 2,4-Nonadienal, (E,E)- | 5910-87-2 | 1 | 3 | 19.03 | 0.02 | - | - | - | - | - | - | - |
| 2,4-Octadienal, (E,E)- | 30361-28-5 | 0 | 0 | 21.67 | 0.01 | - | - | - | - | - | - | - |
| 2,6-Lutidine | 108-48-5 | 2 | 5 | 41.33 | 0.01 | - | - | - | - | - | - | - |
| 2,6-Octadien-1-ol, 3,7-dimethyl-, (Z)- | 106-25-2 | 0 | 0 | 35.66 | 0.02 | - | - | - | - | - | - | - |
| 2,6-Octadiene, 2,6-dimethyl- | 2792-39-4 | 0 | 0 | 21.75 | 0.01 | - | - | - | - | - | - | - |
| 2-Butanol, 2,3-dimethyl- | 594-60-5 | 0 | 0 | 25.78 | 0.03 | - | - | - | - | - | - | - |
| 2-Butanone, 4-phenyl- | 2550-26-7 | 0 | 0 | 25.78 | 0.03 | - | - | - | - | - | - | - |
| 2-Butenal, (E)- | 123-73-9 | 14 | 7 | 64.99 | 0 | - | - | - | - | - | - | - |
| 2-Butenal, 2-methyl- | 1115-11-3 | 0 | 1 | 75.53 | 0 | - | - | - | - | - | - | - |
| 2-Butenal, 3-methyl- | 107-86-8 | 0 | 1 | 48.87 | 0 | - | - | - | - | - | - | - |
| 2-Butenoic acid, ethyl ester, (E)- | 623-70-1 | 0 | 0 | 29.72 | 0.01 | - | - | - | - | - | - | - |
| 2-Decanone | 693-54-9 | 0 | 0 | 21.18 | 0.02 | - | - | - | - | - | - | - |
| 2-Ethylacrolein | 922-63-4 | 0 | 1 | 77.68 | 0 | - | - | - | - | - | - | - |
| 2-Furanmethanol | 98-00-0 | 4 | 6 | 48.27 | 0.01 | - | - | - | - | - | - | - |
| 2-Furanmethanol, 5-methyl- | 3857-25-8 | 0 | 0 | 28.84 | 0.01 | - | - | - | - | - | - | - |
| 2-Heptanol | 543-49-7 | 0 | 0 | 23.49 | 0.01 | - | - | - | - | - | - | - |
| 2-Heptanone | 110-43-0 | 26 | 1 | 45.56 | 0.01 | - | - | - | - | - | - | - |
| 2-Heptanone, 6-methyl- | 928-68-7 | 0 | 0 | 39.99 | 0.01 | - | - | - | - | - | - | - |
| 2-Hepten-1-ol, (E)- | 33467-76-4 | 0 | 0 | 26.46 | 0.01 | - | - | - | - | - | - | - |
| 2-Heptenal, (E)- | 18829-55-5 | 0 | 0 | 37.16 | 0.01 | - | - | - | - | - | - | - |
| 2-Heptenal, (Z)- | 57266-86-1 | 2 | 2 | 40.19 | 0.01 | - | - | - | - | - | - | - |
| 2-Hexanone, 3,4-dimethyl- | 19550-10-8 | 4 | 4 | 35.01 | 0.01 | - | - | - | - | - | - | - |
| 2-Hexenal | 505-57-7 | 0 | 0 | 46.01 | 0.01 | - | - | - | - | - | - | - |
| 2-Octen-1-ol, (E)- | 18409-17-1 | 1 | 3 | 42.12 | 0.01 | - | - | - | - | - | - | - |
| 2-Octenal, (E)- | 2548-87-0 | 1 | 3 | 19.41 | 0.01 | - | - | - | - | - | - | - |
| 2-Pentanone, 3-methyl- | 565-61-7 | 0 | 0 | 22.92 | 0.01 | - | - | - | - | - | - | - |
| (E)-2-Pentenal | 1576-87-0 | 0 | 1 | 50.2 | 0 | - | - | - | - | - | - | - |
| 2-Propenoic acid, 2-methyl- | 79-41-4 | 16 | 33 | 61.55 | 0.01 | - | - | - | - | - | - | - |
| 2-Undecanone | 112-12-9 | 1 | 2 | 17.66 | 0.03 | - | - | - | - | - | - | - |
| 2-Undecenal | 2463-77-6 | 1 | 1 | 39.35 | 0.03 | - | - | - | - | - | - | - |
| 3,6-Octadien-1-ol, 3,7-dimethyl-, (Z)- | 5944-20-7 | 0 | 0 | 41.95 | 0.02 | - | - | - | - | - | - | - |
| 3-Buten-2-ol, 2-methyl- | 115-18-4 | 25 | 49 | 54.58 | 0.01 | - | - | - | - | - | - | - |
| 3-Heptanone | 106-35-4 | 1 | 1 | 68.44 | 0.01 | - | - | - | - | - | - | - |
| 3-Hepten-2-one | 1119-44-4 | 0 | 0 | 30.74 | 0.01 | - | - | - | - | - | - | - |
| 3-Hexen-2-one | 763-93-9 | 0 | 0 | 49.72 | 0.01 | - | - | - | - | - | - | - |
| 3-Octanol | 589-98-0 | 1 | 1 | 30.78 | 0.01 | - | - | - | - | - | - | - |
| 3-Octanone | 106-68-3 | 0 | 0 | 19.48 | 0.01 | - | - | - | - | - | - | - |
| 3-Pentanol | 584-02-1 | 6 | 14 | 79.83 | 0 | - | - | - | - | - | - | - |
| 3-Penten-2-one, (E)- | 3102-33-8 | 0 | 5 | 50.2 | 0 | - | - | - | - | - | - | - |
| 3-Penten-2-one, 4-methyl- | 141-79-7 | 8 | 5 | 50.93 | 0.01 | - | - | - | - | - | - | - |
| 3-Phenylpropanol | 122-97-4 | 0 | 0 | 36.57 | 0.02 | - | - | - | - | - | - | - |
| 3-Pyridinol | 109-00-2 | 0 | 0 | 37.07 | 0.01 | - | - | - | - | - | - | - |
| 4-Ethylcyclohexanol | 4534-74-1 | 0 | 0 | 46.99 | 0.02 | - | - | - | - | - | - | - |
| 4-Octanone | 589-63-9 | 0 | 0 | 19.37 | 0.01 | - | - | - | - | - | - | - |
| 5H-5-Methyl-6,7-dihydrocyclopentapyrazine | 23747-48-0 | 0 | 0 | 34.46 | 0.03 | - | - | - | - | - | - | - |
| 6-Methyl-3,5-heptadiene-2-one | 1604-28-0 | 0 | 0 | 24.05 | 0.01 | - | - | - | - | - | - | - |
| Acetamide, 2-fluoro- | 640-19-7 | 0 | 0 | 49.07 | 0 | - | - | - | - | - | - | - |
| Acetic acid, butyl ester | 123-86-4 | 1 | 4 | 42.65 | 0.01 | - | - | - | - | - | - | - |
| Acetic acid, heptyl ester | 112-06-1 | 1 | 2 | 18.91 | 0.02 | - | - | - | - | - | - | - |
| Acetic acid, hexyl ester | 142-92-7 | 1 | 3 | 13.28 | 0.02 | - | - | - | - | - | - | - |
| Acetic acid, octyl ester | 112-14-1 | 0 | 0 | 18.43 | 0.03 | - | - | - | - | - | - | - |
| Acetic acid, pentyl ester | 628-63-7 | 1 | 3 | 39.66 | 0.01 | - | - | - | - | - | - | - |
| Aciphyllene | 87745-31-1 | 0 | 0 | 22.06 | 0.07 | - | - | - | - | - | - | - |
| a-Methylstyrene | 98-83-9 | 1 | 4 | 46.35 | 0.02 | - | - | - | - | - | - | - |
| a-Phellandrene | 99-83-2 | 17 | 6 | 27.51 | 0.02 | - | - | - | - | - | - | - |
| a-Terpineol | 98-55-5 | 0 | 0 | 29.14 | 0.03 | - | - | - | - | - | - | - |
| Benzaldehyde, 2-methyl- | 529-20-4 | 1 | 3 | 47.28 | 0.02 | - | - | - | - | - | - | - |
| Benzene | 71-43-2 | 0 | 0 | 29.1 | 0.01 | - | - | - | - | - | - | - |
| Benzene, (1-methylethyl)- | 98-82-8 | 1 | 2 | 46.93 | 0.02 | - | - | - | - | - | - | - |
| Benzene, 1-(1,5-dimethyl-4-hexenyl)-4-methyl- | 644-30-4 | 51 | 7 | 19.61 | 0.06 | - | - | - | - | - | - | - |
| Benzene, 1,1'-sulfonylbis[4-chloro- | 80-07-9 | 34 | 2 | 70.57 | 0.1 | - | - | - | - | - | - | - |
| Benzene, 1,2,3-trimethoxy-5-(1-propenyl)-, (E)- | 5273-85-8 | 0 | 0 | 46.72 | 0.06 | - | - | - | - | - | - | - |
| Benzene, 1,2,4,5-tetramethyl- | 95-93-2 | 0 | 1 | 17.74 | 0.03 | - | - | - | - | - | - | - |
| Benzene, 1,2-dimethoxy-4-(1-propenyl)- | 93-16-3 | 84 | 22 | 32.61 | 0.04 | - | - | - | - | - | - | - |
| Benzene, 1-ethenyl-4-methoxy- | 637-69-4 | 0 | 0 | 23.09 | 0.02 | - | - | - | - | - | - | - |
| Benzene, 2-propenyl- | 300-57-2 | 1 | 3 | 39.34 | 0.02 | - | - | - | - | - | - | - |
| Benzene, hexyl- | 1077-16-3 | 0 | 0 | 18.37 | 0.03 | - | - | - | - | - | - | - |
| Benzene, pentyl- | 538-68-1 | 1 | 1 | 34.34 | 0.03 | - | - | - | - | - | - | - |
| Benzene, propyl- | 103-65-1 | 0 | 1 | 47.66 | 0.02 | - | - | - | - | - | - | - |
| Benzenemethanol, a,a,4-trimethyl- | 1197-01-9 | 35 | 10 | 32.26 | 0.03 | - | - | - | - | - | - | - |
| Benzenemethanol, a,a-dimethyl- | 617-94-7 | 1 | 3 | 59.97 | 0.03 | - | - | - | - | - | - | - |
| Benzenepropanoic acid, methyl ester | 103-25-3 | 0 | 0 | 29.05 | 0.03 | - | - | - | - | - | - | - |
| Benzofuran, 2,3-dihydro- | 496-16-2 | 1 | 4 | 50.9 | 0.03 | - | - | - | - | - | - | - |
| Benzoic acid, 3,4,5-trimethoxy-, methyl ester | 1916-07-0 | 0 | 0 | 21.34 | 0.08 | - | - | - | - | - | - | - |
| beta-Myrcene | 123-35-3 | 3 | 2 | 24.96 | 0.02 | - | - | - | - | - | - | - |
| beta-Phellandrene | 555-10-2 | 15 | 4 | 40.3 | 0.02 | - | - | - | - | - | - | - |
| Bicyclo[3.1.1]hept-2-ene, 2,6-dimethyl-6-(4-methyl-3-pentenyl)- | 17699-05-7 | 47 | 10 | 16.23 | 0.09 | - | - | - | - | - | - | - |
| Bicyclo[4.2.0]octa-1,3,5-triene | 694-87-1 | 0 | 0 | 48.07 | 0.03 | - | - | - | - | - | - | - |
| Butanedioic acid, diethyl ester | 123-25-1 | 0 | 0 | 15.96 | 0.03 | - | - | - | - | - | - | - |
| Butanoic acid, 3-methyl-, hexyl ester | 10032-13-0 | 0 | 0 | 18.84 | 0.03 | - | - | - | - | - | - | - |
| Butanoic acid, butyl ester | 109-21-7 | 3 | 2 | 40.73 | 0.02 | - | - | - | - | - | - | - |
| Butanoic acid, ethyl ester | 105-54-4 | 3 | 3 | 54.27 | 0.01 | - | - | - | - | - | - | - |
| Caprolactam | 105-60-2 | 0 | 1 | 54.71 | 0.01 | - | - | - | - | - | - | - |
| Cyclohexane, 1-ethenyl-1-methyl-2,4-bis(1-methylethenyl)-, [1S-(1a,2beta,4beta)]- | 515-13-9 | 0 | 0 | 5.58 | 0.06 | - | - | - | - | - | - | - |
| Cyclohexanone | 108-94-1 | 2 | 3 | 74.99 | 0.01 | - | - | - | - | - | - | - |
| Decanal | 112-31-2 | 1 | 2 | 29.81 | 0.02 | - | - | - | - | - | - | - |
| Decane | 124-18-5 | 0 | 0 | 17.74 | 0.01 | - | - | - | - | - | - | - |
| Decane, 2,9-dimethyl- | 1002-17-1 | 0 | 0 | 9.93 | 0.02 | - | - | - | - | - | - | - |
| Decane, 3-methyl- | 13151-34-3 | 0 | 0 | 17.77 | 0.02 | - | - | - | - | - | - | - |
| Decane, 4-ethyl- | 1636-44-8 | 0 | 0 | 6.02 | 0.02 | - | - | - | - | - | - | - |
| Dimethyl sulfone | 67-71-0 | 76 | 68 | 40.12 | 0 | - | - | - | - | - | - | - |
| Dimethyl trisulfide | 3658-80-8 | 0 | 0 | 10.72 | 0 | - | - | - | - | - | - | - |
| Disulfide, dimethyl | 624-92-0 | 4 | 6 | 39.27 | 0 | - | - | - | - | - | - | - |
| Divinyl sulfide | 627-51-0 | 1 | 4 | 30.48 | 0 | - | - | - | - | - | - | - |
| Dodecane | 112-40-3 | 0 | 0 | 17.74 | 0.02 | - | - | - | - | - | - | - |
| Dodecane, 2,6,11-trimethyl- | 31295-56-4 | 0 | 0 | 14.19 | 0.05 | - | - | - | - | - | - | - |
| Dodecane, 3-methyl- | 17312-57-1 | 0 | 0 | 5.73 | 0.03 | - | - | - | - | - | - | - |
| Dodecane, 6-methyl- | 6044-71-9 | 0 | 0 | 15.19 | 0.03 | - | - | - | - | - | - | - |
| Dodecanoic acid, ethyl ester | 106-33-2 | 2 | 5 | 17.74 | 0.06 | - | - | - | - | - | - | - |
| Estragole | 140-67-0 | 66 | 20 | 36.59 | 0.03 | - | - | - | - | - | - | - |
| Ethanol, 2-butoxy- | 111-76-2 | 0 | 0 | 18.81 | 0.01 | - | - | - | - | - | - | - |
| Ethanone, 1-(1H-pyrrol-2-yl)- | 1072-83-9 | 7 | 8 | 58.37 | 0.01 | - | - | - | - | - | - | - |
| Ethylbenzene | 100-41-4 | 2 | 5 | 49.38 | 0.01 | - | - | - | - | - | - | - |
| Formamide, N,N-dimethyl- | 68-12-2 | 0 | 0 | 25.99 | 0 | - | - | - | - | - | - | - |
| Furan, 2,3-dihydro-5-methyl- | 1487-15-6 | 3 | 6 | 49.49 | 0.01 | - | - | - | - | - | - | - |
| Furan, 2-propyl- | 4229-91-8 | 2 | 4 | 59.19 | 0.01 | - | - | - | - | - | - | - |
| Guanidine | 113-00-8 | 0 | 1 | 24 | 0 | - | - | - | - | - | - | - |
| Hexadecane | 544-76-3 | 1 | 1 | 12.32 | 0.06 | - | - | - | - | - | - | - |
| Hexadecanoic acid, methyl ester | 112-39-0 | 52 | 9 | 18.09 | 0.12 | - | - | - | - | - | - | - |
| Hexanoic acid, ethyl ester | 123-66-0 | 1 | 2 | 22.95 | 0.02 | - | - | - | - | - | - | - |
| Hexanoic acid, methyl ester | 106-70-7 | 1 | 3 | 52.44 | 0.01 | - | - | - | - | - | - | - |
| Methional | 3268-49-3 | 0 | 1 | 53.62 | 0 | - | - | - | - | - | - | - |
| Methyl Isobutyl Ketone | 108-10-1 | 0 | 0 | 17.74 | 0.01 | - | - | - | - | - | - | - |
| Methyl vinyl ketone | 78-94-4 | 18 | 29 | 67.4 | 0 | - | - | - | - | - | - | - |
| Naphthalene, 1,2,3,5,6,7,8,8a-octahydro-1,8a-dimethyl-7-(1-methylethenyl)-, [1S-(1a,7a,8aa)]- | 10219-75-7 | 51 | 14 | 34.6 | 0.08 | - | - | - | - | - | - | - |
| Neodecanoic acid | 26896-20-8 | 44 | 7 | 12.96 | 0.08 | - | - | - | - | - | - | - |
| Nonanal | 124-19-6 | 5 | 5 | 40.28 | 0.02 | - | - | - | - | - | - | - |
| Nonanoic acid, ethyl ester | 123-29-5 | 1 | 2 | 36.62 | 0.03 | - | - | - | - | - | - | - |
| Octanal | 124-13-0 | 0 | 0 | 19.07 | 0.01 | - | - | - | - | - | - | - |
| Octane, 4-ethyl- | 15869-86-0 | 0 | 0 | 16.04 | 0.01 | - | - | - | - | - | - | - |
| Octanoic acid, methyl ester | 111-11-5 | 1 | 2 | 18.71 | 0.02 | - | - | - | - | - | - | - |
| Oxepine, 2,7-dimethyl- | 1487-99-6 | 0 | 0 | 38.95 | 0.02 | - | - | - | - | - | - | - |
| p-Cymene | 99-87-6 | 1 | 2 | 27.2 | 0.02 | - | - | - | - | - | - | - |
| Pentanal | 110-62-3 | 0 | 1 | 59.53 | 0 | - | - | - | - | - | - | - |
| Pentanal, 3-methyl- | 15877-57-3 | 0 | 0 | 37.56 | 0.01 | - | - | - | - | - | - | - |
| Phenylethyl Alcohol | 60-12-8 | 5 | 6 | 44.03 | 0.02 | - | - | - | - | - | - | - |
| Propane, 2-ethoxy- | 625-54-7 | 4 | 8 | 34.27 | 0 | - | - | - | - | - | - | - |
| p-Xylene | 106-42-3 | 12 | 12 | 48.74 | 0.01 | - | - | - | - | - | - | - |
| 2,5-Dimethylpyrazine | 123-32-0 | 2 | 6 | 34.12 | 0.01 | - | - | - | - | - | - | - |
| 2,6-Dimethylpyrazine | 108-50-9 | 2 | 5 | 35.27 | 0.01 | - | - | - | - | - | - | - |
| Methylpyrazine | 109-08-0 | 0 | 0 | 27.1 | 0.01 | - | - | - | - | - | - | - |
| Pyrazine, tetramethyl- | 1124-11-4 | 9 | 4 | 20.01 | 0.03 | - | - | - | - | - | - | - |
| Pyrazine, trimethyl- | 14667-55-1 | 1 | 3 | 32.27 | 0.02 | - | - | - | - | - | - | - |
| Pyridine | 110-86-1 | 0 | 0 | 42.32 | 0.01 | - | - | - | - | - | - | - |
| Pyridine, 3-ethyl- | 536-78-7 | 0 | 0 | 20.53 | 0.01 | - | - | - | - | - | - | - |
| Pyridine, 3-methyl- | 108-99-6 | 11 | 6 | 73.75 | 0.01 | - | - | - | - | - | - | - |
| Pyrrole | 109-97-7 | 0 | 0 | 27.75 | 0 | - | - | - | - | - | - | - |
| Terpinen-4-ol | 562-74-3 | 4 | 3 | 40.75 | 0.03 | - | - | - | - | - | - | - |
| Thiophene | 110-02-1 | 1 | 2 | 31.22 | 0 | - | - | - | - | - | - | - |
| Thiophene, 2-methyl- | 554-14-3 | 2 | 3 | 35.85 | 0.01 | - | - | - | - | - | - | - |
| Thiophene, 3-methyl- | 616-44-4 | 1 | 1 | 84.17 | 0.01 | - | - | - | - | - | - | - |
| Toluene | 108-88-3 | 9 | 16 | 42.58 | 0.01 | - | - | - | - | - | - | - |
| trans, trans-nona-2,4-dienol | 64576-90-5 | 0 | 0 | 27.45 | 0.02 | - | - | - | - | - | - | - |
| trans-beta-Ocimene | 3779-61-1 | 0 | 0 | 15.06 | 0.02 | - | - | - | - | - | - | - |
| Tridecane | 629-50-5 | 0 | 0 | 17.89 | 0.03 | - | - | - | - | - | - | - |
| Tridecane, 2-methyl- | 1560-96-9 | 0 | 0 | 5.75 | 0.04 | - | - | - | - | - | - | - |
| Tridecane, 3-methyl- | 6418-41-3 | 0 | 0 | 5.24 | 0.04 | - | - | - | - | - | - | - |
| Undecanal | 112-44-7 | 0 | 0 | 22.9 | 0.03 | - | - | - | - | - | - | - |
| Undecane | 1120-21-4 | 0 | 0 | 17.15 | 0.02 | - | - | - | - | - | - | - |
| Undecane, 2-methyl- | 7045-71-8 | 0 | 0 | 17.8 | 0.02 | - | - | - | - | - | - | - |
| Undecane, 4,6-dimethyl- | 17312-82-2 | 0 | 0 | 13.03 | 0.03 | - | - | - | - | - | - | - |
| Undecane, 4,7-dimethyl- | 17301-32-5 | 0 | 0 | 13.58 | 0.03 | - | - | - | - | - | - | - |
| Urethane | 51-79-6 | 0 | 0 | 3.97 | 0 | - | - | - | - | - | - | - |

Note: The four annotated active pharmaceutical ingredients were highlighted with red words; These 38 differential volatile metabolites revealed by OPLS-DA analysis were highlighted by yellow.
